# Enhanced surface water-energy coupling on the Tibetan Plateau over the past six decades (1960–2020)

**DOI:** 10.1016/j.fmre.2023.12.006

**Published:** 2024-01-05

**Authors:** Kun Yang, Jing Sun, Hui Lu, Kaighin A. McColl, Yaozhi Jiang, Qing He

**Affiliations:** a Ministry of Education Key Laboratory for Earth System Modeling, Department of Earth System Science, Tsinghua University, Beijing 100084, China; b National Tibetan Plateau Data Center, State Key Laboratory of Tibetan Plateau Earth System Science, Environment and Resources, Institute of Tibetan Plateau Research, Chinese Academy of Sciences, Beijing 100101, China; c Department of Earth and Planetary Sciences, Harvard University, Cambridge, MA 02138, USA; d School of Engineering and Applied Sciences, Harvard University, Cambridge, MA 02138, USA; e River and Environmental Engineering Laboratory, The University of Tokyo, Tokyo 113 8656, Japan

**Keywords:** Tibetan Plateau, Evaporative fraction, Water-energy coupling, Surface flux equilibrium, Climate change, Spatial pattern

## Abstract

Long-term changes in surface water and energy budgets on the Tibetan Plateau (TP) are essential for understanding the variability of the Asian summer monsoon and water resources. The water budget and energy budget are inherently coupled, and in this study evaporative fraction (EF, the proportion of the surface available energy used for evapotranspiration) is used to quantify their coupling strength and simultaneously study its response to climate change in the TP. A so-called ‘surface flux equilibrium’ (SFE) method that uses only air temperature and humidity as inputs to estimate EF is evaluated and adopted for understanding EF variability in the TP during the warm season of the past six decades (1960–2020). The results show an overall increase in EF of the TP, but this increase is not uniform, instead comprising three distinct stages of change, i.e., little change until the 1980s, a significant increase in the next two decades, and a slight but insignificant decrease in the last two decades. Accordingly, evapotranspiration has undergone similar decadal changes over this period. More importantly, there is a spatial pattern of the response to climate change, showing a stronger enhancement of EF in the west TP than in the east TP during 1979–2020. This response pattern shows a weakened contrast in the surface water cycle between the dry west and the wet east TP. The enhanced coupling and diminished spatial contrast may provide a perspective for understanding spatiotemporal changes in environment and ecology. Finally, it is worth pointing out that given the simplicity of the SFE method, it is expected that it can be applied in climate-sensitive regions outside the TP.

## Graphical abstract

EF has higher values in the east than in the west of the Tibetan Plateau (a), but the EF increased more in the west than in the east (b).

**Figure:**
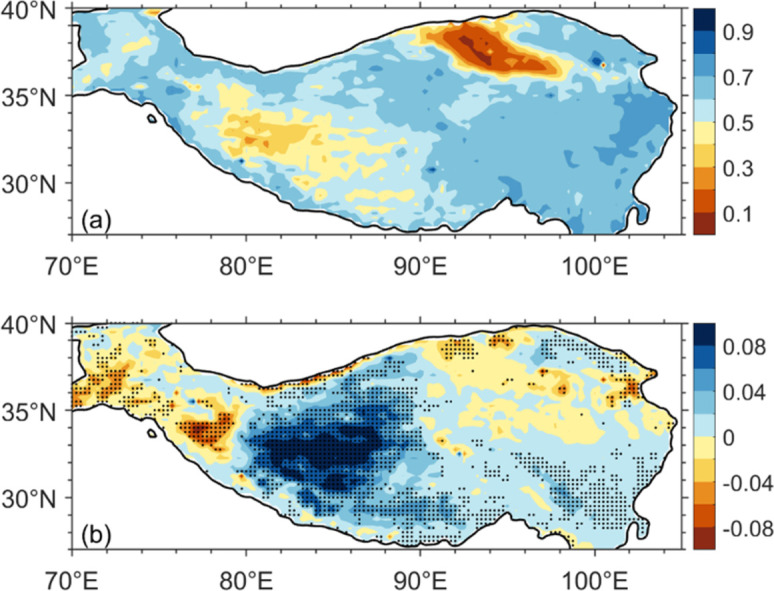

## 1 Introduction

The Tibetan Plateau (TP) exerts strong thermal forcing on Asian summer monsoon circulations and provides abundant water resources for its surroundings. It is crucial to understand changes in the TP surface energy budget and water budget that modulate the variability of the monsoon circulations and the availability of the water resources [1]. The TP has undergone rapid warming since the mid-1950s [2] and moistening since the 1980s [3], which in turn have reshaped the TP environment, altered ecosystems and threatened infrastructure [[3], [4], [5], [6], [7]]. Particularly, the change in precipitation exhibits a distinct spatial pattern, less variable or even decreasing since the mid-1990s in the monsoon-influenced southern and eastern margin, while increasing in the central and western parts of the TP [[3], [8], [9]]. It is critical to understand how the surface energy budget and water budget have responded to climate change. Studies on the surface energy and water budgets on the TP have been focused on surface sensible heat flux (SH) and evapotranspiration (ET), and significant progress has been achieved in understanding their changes since the 1980s, when reanalysis data are widely available. That is, the surface sensible heat has weakened [[10], [11], [12]], while ET has increased [[3], [13], [14], [15]] in response to the warming and moistening over the TP. However, their long-term changes (prior to 1980) are little known, due to limitations of data and methods used in previous studies.

The surface water budget and energy budget are inter-dependent and their coupling may provide a new perspective to understand their long-term changes simultaneously. In most studies, trends in surface sensible heat were derived from station data using empirical formulas (e.g. [10]), while trends in ET were estimated through a variety of methods, such as water balance [[16], [17]], the maximum entropy production [13], land hydrological modeling [3] or satellite remote sensing [[15], [18]]. There are two limitations in these studies. First, these studies need high-quality data of multiple meteorological inputs, which are usually not available for a long period. This explains why the changes in surface energy budget and water budget prior to the 1980s are seldom investigated. Second, the surface water budget and energy budget are coupled inherently through ET or latent heat flux, but these studies derived the changes in energy budget and water budget under different frameworks or methods, which may cause contradictory results (see Discussion). Therefore, alternative methods for investigating the water-energy coupling should be sought to avoid these limitations in the inputs and methods.

In this study, the Evaporative Fraction (EF), which is the proportion of the surface available energy used for ET, is used as a measure of the surface water-energy coupling strength. EF is modulated by soil moisture and vegetation [[19], [20], [21]]. Conventionally, EF is quantified with observed or estimated ET and available energy, but it is particularly difficult to obtain long-term estimates of ET and available energy due to the lack of reliable inputs.

In recent years, new approaches have been developed to calculate surface fluxes and EF with observation data from routine weather stations. One approach is the ETRHEQ (ET from Relative Humidity (RH) at Equilibrium) developed by Salvucci and Gentine [22]. A key step to estimate ET is the estimation of the surface conductance to water vapor transport (or surface soil evaporation resistance and stomatal resistance), which is controlled by hydrologic and biological conditions of the surface. In the ETRHEQ, the daily-constant surface conductance is obtained through minimizing the vertical variance of the RH profile within the surface boundary layer averaged over the course of the day. After introducing some empirical formulas, Rigden and Salvucci [23] extended the application of ETRHEQ to weather stations with hourly measurements of air temperature, humidity, wind speed, pressure, and downwelling shortwave radiation. McColl et al. [24] presented a theoretical basis for ETRHEQ, arguing that, at steady state, “ETRHEQ is equivalent to assuming a balance between the surface heating and moistening terms in the steady-state relative humidity budget”. They called this state “surface flux equilibrium” (SFE), and derived a simpler expression for EF based on it [[25], [26]]. In particular, the SFE equation for EF requires zero free parameters, no land surface or wind speed information, and, unlike the early ETRHEQ approach, does not require information on vegetation height (which ETRHEQ uses to parameterize aerodynamic resistance). The only required inputs are air temperature and humidity. This approach is applicable to calculate daily to monthly surface fluxes and EF. While simpler than other approaches to estimating evapotranspiration, SFE estimates are about as accurate as those from state-of-the-art eddy covariance observations [25].

This study aims to elucidate the long-term changes in surface water-energy coupling (denoted by EF) and its spatial pattern on the TP. The period of interest is from the 1960s, since when the number of weather stations on the TP has become stable. We adopted the SFE approach because its inputs (air temperature and humidity) have been measured at the weather stations. We first validate the performance of the SFE approach in reproducing the decadal variation of EF in the TP. Then, a long-term time series of EF is constructed using station data and its decadal variation is analyzed. Third, this station data-based EF time series is used to test the applicability of ERA5 data in reproducing the decadal changes of EF, based on which the ERA5 data are used to show the spatial pattern of EF response to the climate change over the TP. Finally, we discuss the cause for the long-term variations of EF and the decadal change relationship between EF and ET.

## 2 Method and data

### 2.1 SFE-based approach for EF calculation

EF is defined as:

(1) $$\mathrm{EF}\;=\;\frac{\lambda E}{\lambda E+H},$$

where H (W·m^−2^) is surface sensible heat flux; λE (W·m^−2^) is surface latent heat flux, with λ (2*.*5008 × 10^6^ J·kg^−1^) and E (kg·m^−2^·s^−1^) being the latent heat of vaporization of water and the evapotranspiration, respectively.

According to McColl et al. [24], in regions where external water vapor transport is not strong, there is a balance between the surface heating and moistening terms in the steady-state RH budget within the boundary layer on a daily-to-monthly time scale. In these regions, the SFE-based approach yields the Bowen ratio (the ratio of sensible heat flux to latent heat flux) and EF as follows:

(2a) $$B=\frac{R_{v}c_{p}T_{a}^{2}}{\lambda^{2}q_{a}},$$

(2b) $$\mathrm{EF}\;=\;\frac{1}{1+B},$$

where B is Bowen ratio, $R_{v}$ (461 J·kg^−1^·K^−1^) is the gas constant for water vapor, $c_{p}$ (1004 J·kg^−1^·K^−1^) is the specific heat of air at constant pressure, $q_{a}$ (kg^−1^) and $T_{a}$ (K) are near-surface specific humidity and air temperature.

McColl and Rigden [25] have demonstrated the validity of Eq. 2 at flux stations at a global scale, and He et al. [27] have used it to characterize land aridity. As the input variables of Eq. 2 are routinely measured at weather stations, it can be widely used to calculate long-term EF.

### 2.2 Station data and reanalysis data

**Fig. 1:**
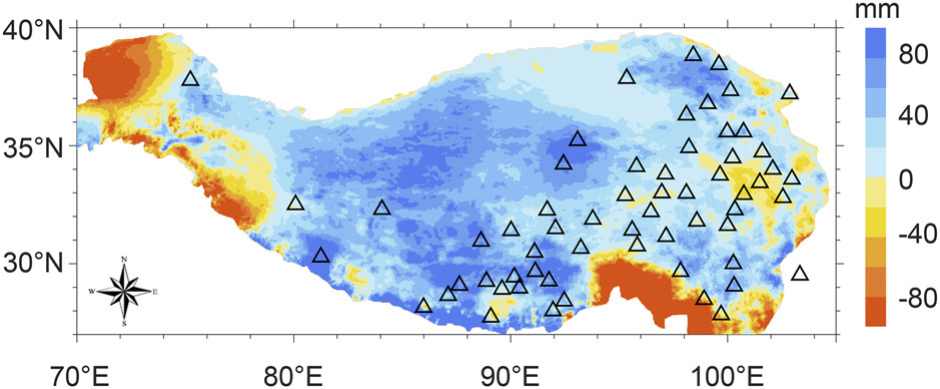
**Distribution of 61 weather stations (triangles) that have long-term (1960–2020) observations on the Tibetan Plateau.** The color shows the precipitation difference between the two periods before 1995 (1979–1995) and after (1996–2020) (the latter minus the former) during the warm season over the TP, derived from a high-resolution precipitation dataset for the TP (Jiang et al., 2023).

The TP climate varies greatly in space and time. During the warm season (from May to September), precipitation gradually decreases from southeast TP to northwest TP, mainly due to the intrusion of South Asian monsoon from the southeast; during the cold season, precipitation occurs mainly in the mountainous areas surrounding the TP and the interior of the TP is dry. Since the mid-1990s, atmospheric circulation anomalies have led to overall moistening of the plateau [28], resulting in the expansion of many lakes [[29], [30]]. Fig. 1 shows the precipitation difference during the warm season between the two periods before and after 1995, which is derived from a high-resolution precipitation dataset for the TP developed by Jiang et al. [31].

Observed monthly data of air humidity and temperature from weather stations are used to estimate EF with the SFE approach. As shown in Fig. 1, there are 61 weather stations on the TP with data covering the period 1960–2020. The measured air humidity and temperature data have stable quality and temporal consistency, and thus are suitable for analyzing long-term changes. In addition, precipitation data collected at these stations are used in this study to support the analysis of EF.

In addition, monthly surface sensible and latent heat fluxes, air temperature, specific humidity, and precipitation from ERA5 are used to support our analysis. ERA5 is the ECMWF latest generation of atmospheric reanalysis data [32]. The reanalysis system has assimilated air temperature and humidity data from weather stations. Since 1979 to date, the system has assimilated a large amount of satellite data, resulting in a more reliable reanalysis quality after 1979 than before 1979.

The station data are used to investigate the long-term changes in EF with the support of the SFE theory. The use of ERA5 has threefold purposes. First, given that ERA5 can provide both high-quality inputs of SFE approach and data of surface sensible/latent heat fluxes since 1979, it is useful to compare its EF values with corresponding SFE estimates. Second, given no satellite data assimilated in ERA5 before 1979, the change in EF of ERA5 for the early period (1960–1978) is evaluated through comparison with the SFE approach and station data-based results. Third, ERA5 is used to analyze the spatial pattern of EF changes in the TP.

## 3 Results

### 3.1 Comparison of SFE with ERA5 on the Tibetan Plateau

In this section, we compare EF estimated using SFE with corresponding estimates from ERA5 over the TP. SFE is most applicable to regions in which convergence of moisture and heat are small. While that approximation is almost never exactly satisfied, the errors induced by this deviation, across a broad range of locations, are roughly similar to standard eddy covariance measurement errors [25]. Still, the TP is subject to strong advection, so it is prudent to first compare the SFE EF estimates to another EF estimate. Ideally, we would use long-term surface sensible and latent heat flux observations, but these observations are lacking on the TP. Alternatively, ERA5 has assimilated a large amount of satellite data for this period, and the surface heat flux data and near-surface air temperature and humidity data are approximately self-consistent in ERA5 for 1979–2020 and thus may offer a useful comparison with the SFE estimates. That is, EF can be calculated from Eq. 1 with ERA5 surface heat flux data or estimated from Eq. 2 (i.e. the SFE approach) with ERA5 near-surface air temperature and humidity data, respectively. If the decadal changes between the two EF estimates are similar, this provides some support for the use of SFE in analyzing decadal variations of EF.

**Fig. 2:**
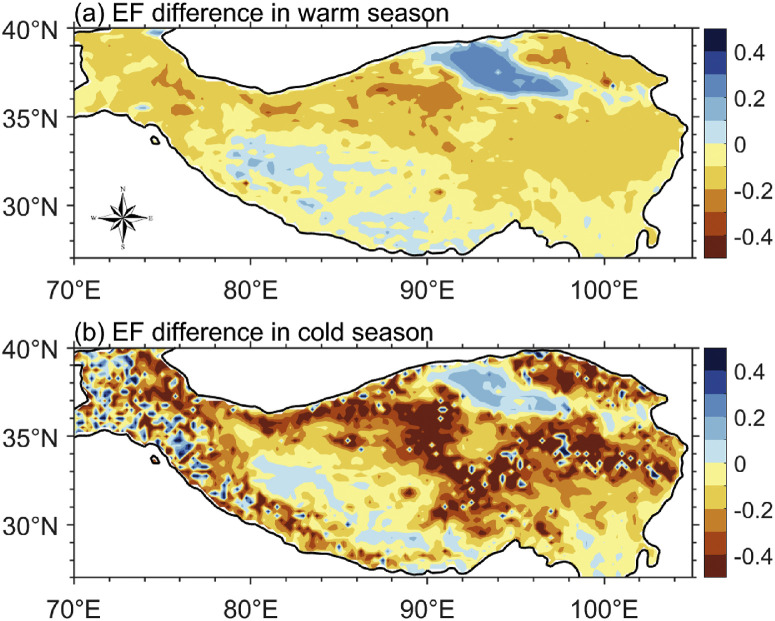
**The spatial distribution of the differences in EF calculated by two approaches** (Eq. 1**and**Eq. 2) **(the latter minus the former) for the period 1979–2020.** (a) during the warm season (May-September), (b) during the cold season (October-April). Eq. 1 uses surface sensible and latent heat flux data from ERA5, and Eq. 2 uses near-surface air temperature and specific humidity from ERA5.

Fig. 2 compares the spatial distribution of the differences in EF calculated by the two approaches for the period 1979–2020. The EF differences in Fig. 2a are the average values during the warm season (May-September) when evapotranspiration is strong, and those in Fig. 2b are for the cold season (October-April) when evapotranspiration is weak. As can be seen, the differences calculated by the two approaches are overall small in the warm season, but they are large in the cold season. In the cold season, westerly winds are strong, and thus it is difficult to reach local equilibrium between surface fluxes and RH, so the EFs calculated by the two approaches are very different. Therefore, in the following, we only analyzed the case in the warm season.

Even in the warm season, Fig. 2a shows that the two approaches yield different EF values. In most regions of the TP, the EF calculated from surface heat fluxes (Eq. 1) is generally larger than that calculated from the SFE approach (Eq. 2). However, in the two arid regions (Southwest TP and Qaidam), the EF calculated from surface heat fluxes is generally less than the EF calculated from SFE. These results are broadly consistent with previous studies, which also found that SFE tends to somewhat overestimate EF in dry regions or periods, and underestimate it in wet regions or periods [[25], [26]]. However, it is worth noting that the differences between the ERA5 estimates and the SFE estimates are not necessarily due to errors in the SFE estimates. Indeed, Chen et al. [26] found that SFE estimates were more accurate than a reanalysis, although they used a different reanalysis to that used here.

**Fig. 3:**
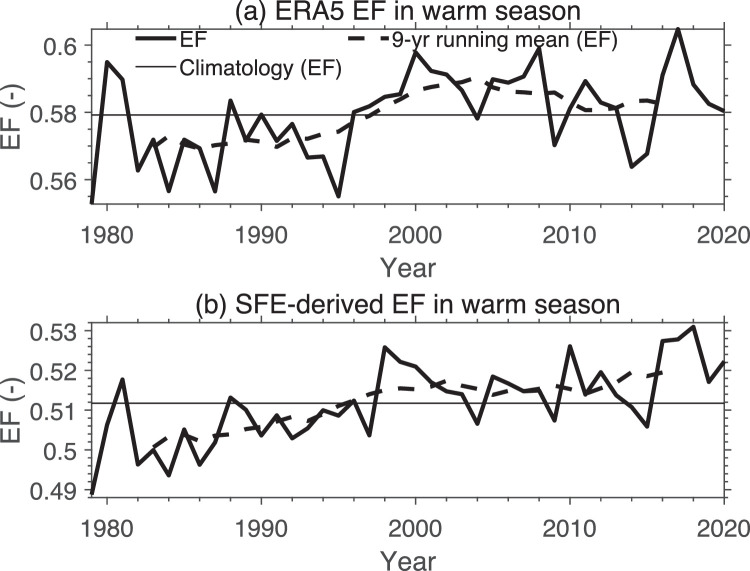
**Inter-annual variation of EF averaged over the Tibetan Plateau during the warm (May-September) season from 1979 to 2020.** (a) Calculated with Eq. 1 using surface sensible and latent heat flux data from ERA5, (b) calculated with the SFE approach using near-surface air temperature and specific humidity from ERA5 as inputs. The horizontal solid lines are the mean over the whole period, and the dashed lines are the 9-year running mean to show the decadal variation.

Our particular focus is whether the SFE approach can be used to calculate long-term changes in EF. Fig. 3 shows changes in the plateau-averaged EF during the warm season of 1979–2020 estimated by the surface heat flux calculation and the SFE approach, respectively. Again, the input surface heat fluxes and near-surface meteorological data are from ERA5. Despite the systematic differences between the two, they show similar inter-decadal variability (Fig. 3). Therefore, even if differences between the two estimates are solely attributed to errors in SFE, it seems reasonable to conclude that the long-term variation of EF can be estimated reasonably with SFE in the TP region.

### 3.2 Long-term change in EF during the warm season

**Fig. 4:**
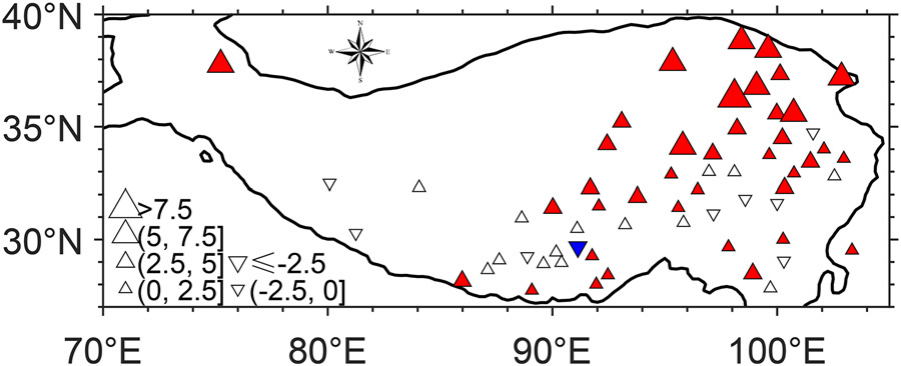
**EF linear trend (10^−4^ yr^−1^) during 1960–2020 at each station on the Tibetan Plateau for the warm season.** EF is estimated by the SFE approach with station data as input. Solid symbols denote passing significance test of *p* < 0.05. Red color denotes increased EF, and blue colors denotes decreased EF.

In this section we use station data to reveal the change in EF over the period 1960–2020. Meanwhile, we investigate how well ERA5 can reproduce the change. The air temperature and humidity data since 1960 from 61 CMA stations are used as inputs to the SFE approach. Fig. 4 shows the linear trend in the calculated EF at each station during the warm season since 1960. It can be seen that most of the stations see an increasing trend, and in the north the EF has increased more.

**Fig. 5:**
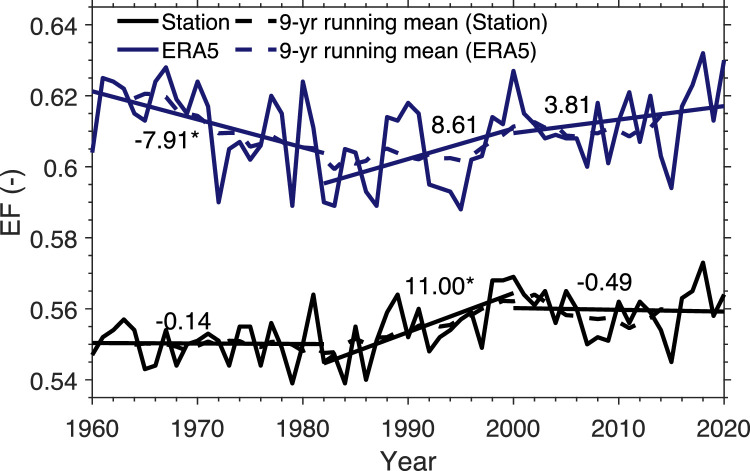
**Station-averaged inter-annual variation of EF for the warm season of 1960–2020 on the TP, estimated by the SFE approach using station data as input data.** For comparison, the variation of EF derived from the ERA5 surface flux data, averaged over the grids collocated with the stations, is given. The dashed lines are the 9-year running mean to show the decadal variation. The numbers represent the trend (unit: 10^−4^ yr^−1^). * indicates passing significance test of *p* < 0.05.

How different are the station data-based EF estimates from the ERA5 estimates? Fig. 5 shows the station-averaged inter-annual variations of EF derived from the station data and EF from ERA5 fluxes. It can be found that EF derived from the station data is lower than that derived from ERA5 flux data. The EF derived from the station data has little inter-decadal oscillation before the mid-1980s and increases significantly during the following two decades. This inter-decadal increase is corresponding to the wetting processes since the mid-1990s [28]. After 2000, EF stops increasing and even shows a slight decline. The EF variation derived from ERA5 surface flux data resembles that from the station data, but shows a different trend before the period of mid-1980s. That is, ERA5 shows a decreasing trend before the 1980s with the maximum occurring in 1960s, but this decreasing trend is not seen in the EF derived from the station data. Instead, the latter yields the minimum EF during the 1960s.

**Fig. 6:**
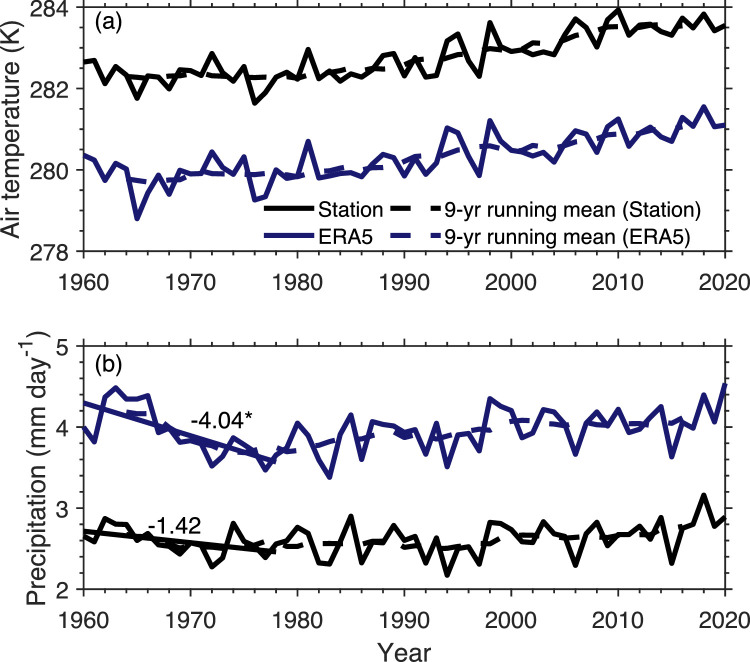
**Inter-annual variation of air temperature (a) and precipitation (b) on the Tibetan Plateau during the warm season (May-September) of 1960–2020, averaged over stations and ERA5 collocated grids.** The dashed lines are the 9-year running mean to show the decadal variation. The numbers represent the trend (10^−2^ mm day^−1^ yr^−1^). * indicates passing significance test of *p* < 0.05.

The meteorological data at the stations are used as indirect evidence to show that ERA5-derived EF is questionable before the 1980s. We compared the time series of air temperature and precipitation between the station observations and ERA5 reanalysis data during the warm season, as shown in Fig. 6. Air temperature from the station data and ERA5 data show similar variations during the whole 1960–2020 period. However, different variations of precipitation between the two datasets can be seen clearly before the 1980s. ERA5 shows a strong decrease in precipitation before the 1980s, but such a strong trend is not observed at the stations. This explains why ERA5-derived EF has a false decreasing trend before the 1980s.

It is not surprising that ERA5 cannot produce the precipitation and EF changes before the 1980s. The merit of ERA5 is its assimilation of multiple sources of data, including data from land stations (surface pressure, air temperature, RH and snow depth) and satellite data [32]. The latter is a dominant data source, particularly for the TP where station data are sparse. Before the 1980s (pre-satellite era), the lack of satellite data assimilation may make the reanalysis data less reliable, which may have distorted the surface water and energy budgets in the reanalysis.

In summary, EF has been enhanced in the TP during the warm season over the past six decades, with little change before the 1980s, a significant increase during the next two decades and a slight decline in last two decades. ERA5 can reproduce the inter-decadal trend of EF consistent with the in-situ observations since the 1980s but cannot do so for earlier decades.

### 3.3 Spatial pattern of EF changes

**Fig. 7:**
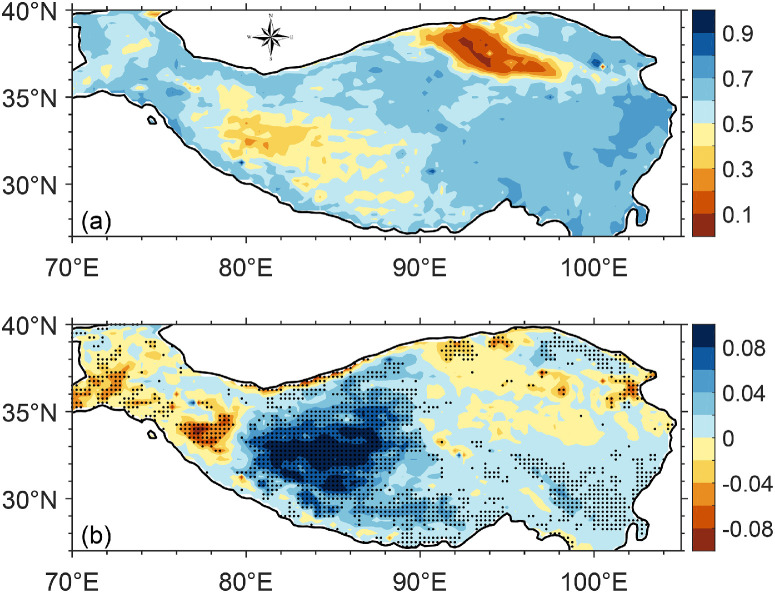
**The distribution of (a) the EF climatology over the period 1979–2020 and (b) EF difference between the two periods before 1995 (1979–1995) and after 1995 (1996–2020) (the latter minus the former) in the warm season in the TP, derived from the land fluxes of ERA5.** The dot in (b) indicates areas passing significance test of *p* < 0.05.

The eastern and western parts of the TP have completely different climates. The east is much wetter than the west, and thus the EF in the east is much greater than in the west (Fig. 7a). Earlier studies have shown that precipitation on the TP has increased since the mid-1990s, and since then, the water cycle has changed considerably. How does the spatial pattern of surface water-energy coupling change over the past decades in the context of a warming and moistening climate? There are few stations in the western TP, so it is not possible to investigate the spatial pattern of the EF change through station data. Nevertheless, Section 3.2 has shown that ERA5 can approximately reproduce the decadal variations in EF since the era of satellite data assimilation. Thus, the ERA5 data were used to investigate the EF differences between the eastern and western TP during the recent four decades.

Fig. 7b shows the distribution of the EF difference between the two periods before 1995 (1979–1995) and after (1996–2020) (the latter minus the former) in the warm season over the TP. The changes are much stronger in the western TP, particularly in the Qiangtang Plateau (the region of about 80˚−90˚ E, 30˚−36˚ N). That is, the plateau moistening has significantly enhanced the water-energy coupling in the west. This response pattern has reduced the EF gradient between the eastern and western TP over the past 40 years.

It can be noted that the linear trend in EF from 1960 to 2020 at most stations on the western TP did not pass a significance test (Fig. 4), which seems inconsistent with the results in Fig. 7b. In actuality, the stations in the western TP are very sparse and are not located in the core region where EF increased significantly. The inconsistency between Figs. 4 and 7b indeed occurs for the Gaize station (at 84.1˚ E, 32.3˚ N), which is situated in the core region. Many studies have shown that most lakes in this region have expanded considerably over the last 40 years. However, the lake closest to the Gaize station (Lagkor Co) has only experienced slight expansion, which corresponds well with the small change in precipitation at this station. In other words, the precipitation change and thus the EF change at this station are too small to represent the regional wetting. This issue was specifically demonstrated in an early study (Fig. 2a-b in Sun et al., 2020). Therefore, there is no substantial discrepancy between the station-derived EF change (Fig. 4) and the ERA5-derived one (Fig. 7b).

## 4 Discussion

### 4.1 Causes for the inter-decadal variability in EF

Fig. 5 in Section 3.2 illustrates three distinct stages of inter-decadal variability in the warm-season EF from 1960 to 2020. As EF highly depends on soil wetness, whose temporal change is primarily influenced by precipitation, we discussed how precipitation change influenced the inter-decadal variability in EF.

**Fig. 8:**
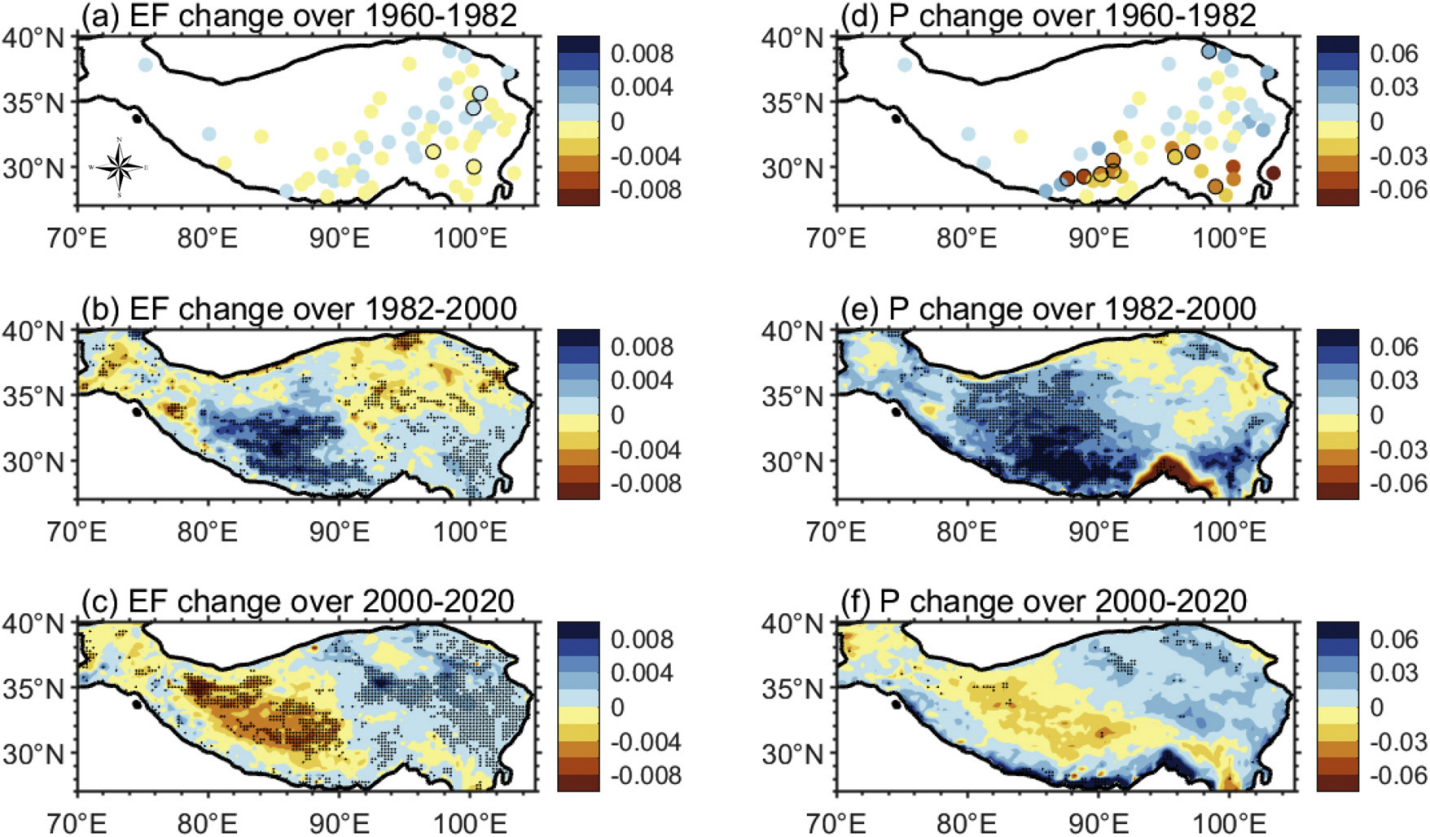
**Linear changes in EF (a-c) and precipitation (d-f) during the warm season of three periods:** (a, d) 1960–1982, (b, e) 1982–2000, (c, f) 2000–2020. (a, d) derived from station data, (b, e) and (c, f) derived from ERA5 data. The unit is yr^−1^ for EF change and mm day^−1^ yr^−1^ for precipitation change. The dotted area indicates passing significance test of *p* < 0.05.

Fig. 8 shows the spatial distribution of EF and precipitation changes during the three stages since 1960, with Fig. 8a, 8d derived from station data and Fig. 8b-c, 8e-f derived from ERA5 data that is relatively reliable since 1979. In general, precipitation changes and EF changes are approximately consistent in space. During 1960–1982, precipitation either increased or decreased, depending on the location, resulting in small changes in spatially averaged EF. During 1982–2000, most of the TP, especially the Qiangtang Plateau, saw a significant increase in precipitation, leading to a significant increase in EF. During 2000–2020, a slight increase in precipitation on the eastern TP led to an increase in EF in the region, while a slight decrease in precipitation on the Qiangtang Plateau led to a decrease in EF in the region; the increase and decrease in EF offset each other, resulting in an insignificant change in average EF. Overall, the post-2000 average of EF is significantly higher than the pre-1980 value.

### 4.2 Decadal correlation between EF and ET

The TP is called Asian Water Tower, for it is the source regions of major rivers of Asia. As a major component of surface water balance, ET is highly variable in both space and time. With the overall warming and moistening of the TP, decadal changes in ET on the TP have received much attention. ET is typically estimated through remote sensing or land surface modeling, which require the input of a number of meteorological data that are difficult to obtain. However, the SFE approach can easily estimate the decadal change in EF based on near-surface air temperature and humidity data that are widely available. It is attractive if the decadal changes in ET can be indicated by the decadal changes in EF. This issue is discussed below.

**Fig. 9:**
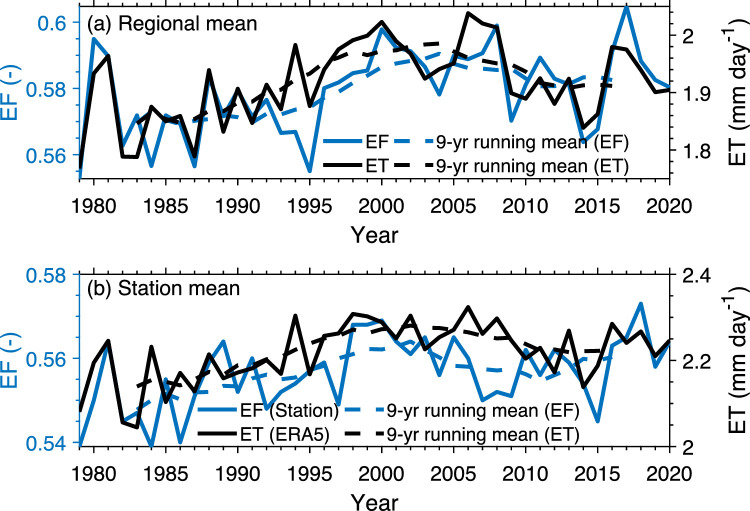
**Inter-annual variation of EF and ET in the TP during the warm season of 1979–2020.** (a) EF and ET of ERA5 derived from regional mean, (b) EF derived from station data and ET of ERA5 derived from the collocated pixel data. The dashed lines are the 9-year running mean to show the decadal variation.

Fig. 9a shows the inter-annual variation of ERA5 EF and ERA5 ET in the TP during the warm season of 1979–2020, and Fig. 9b shows the variation of station data-based EF and ERA5 ET. It can be seen that the EF and ET have a weak correlation on the inter-annual scale, but both show positive linear trends over the period 1979–2020 and exhibit decadal changes (denoted by the line of nine-year running mean), with an increase before about 2000 and a smaller decrease afterwards. The correlation coefficient between EF and ET of ERA5 is 0.88 (*p* < 0.05) on decadal scale. This high correlation is even found between station data-based EF and ERA5 ET, for which the correlation coefficient is 0.9. Given this strong positive correlation between EF and ET on the decadal scale, the decadal changes in ET can be roughly indicated by EF, which can be estimated from observations of near-surface air temperature and specific humidity alone.

We compared the changes in ET between this study and early studies. SFE-derived trend in EF since 1980 is positive. The increase of ET has been shown in early studies. For example, Zhang et al. [16] indicated the increase during 1966–2001 through water balance estimation for 16 TP basins, and Yang et al. [3] showed an increase for the period 1984–2006 through land surface modeling at CMA stations. For a longer period (from the 1980s to about 2020), recent studies presented overall positive trends in ET through remote sensing [[15], [18], [33]], the complementary relationship for ET estimation [[34], [35], [36]]), and land surface modeling [37]. All these results are consistent with the SFE-derived one.

In contrast to the overall consistency of the long-term trend among these studies, they diverge on the change in ET over the more recent two decades (since 2000). Wang et al. [35] showed a positive trend since 2000, Ma and Zhang [15] showed a negligible negative trend since 2000, but more studies presented a decline in ET (see Table 1 in [34]). As shown in Fig. 9, the ET change derived from the SFE is sensitive to the period of concern. For a shorter period (2000–2010), there is a large negative trend, as consistent with Song et al. [38] and Zhang et al. [36]; for a longer period (2000–2020), there is a small negative trend, as consistent with Han et al. [39] and Wang et al. [34].

In summary, the SFE approach can easily calculate EF with limited input. The estimated EF provides an independent view of decadal changes in ET, which are quite consistent with the results of most previous studies.

## 5 Concluding remarks

The TP has experienced significant climate changes over the past 60 years, leading to changes in the cryosphere and water cycle. Particularly, atmospheric circulation anomalies around the TP have caused more precipitation in the central and western TP [[28], [40]] and decadal dry/wet oscillation in the southeastern TP [41] over the past four decades. This study investigates the response of water-energy coupling to the climate change. This coupling between water and energy is characterized by EF.

We first show the applicability of a new approach based on local surface flux equilibrium (SFE) proposed by McColl et al. [24]. This approach requires only daily-to-monthly air temperature and specific humidity inputs to estimate EF. The EF calculated by this approach for the Tibetan Plateau tends to be lower than reality, but this approach is able to estimate the decadal variability of EF. The EF calculated using long time series of station data shows that there is an increasing trend in water and energy coupling on the Tibetan Plateau over the past 60 years. This trend is not uniform but includes three distinct stages of change with a pronounced increase occurring from the mid-1980s to 2000, little decadal change until the 1980s and a slight decline after 2000. The three stages correspond well with the inter-decadal changes in precipitation. The latest reanalysis data ERA5 can reproduce the increasing trend of EF, but gives a decreasing trend before the 1980s, which is not consistent with station data. This problem may stem from the scarcity of satellite data assimilated in ERA5 during the early decades. Leveraging the ability of ERA5 to reflect EF changes since the era of satellite data assimilation, we found that the EF increase in the western TP is much larger than that in the eastern TP. The greater increase in EF in the west relative to EF in the east suggests a narrowing of the east-west contrast in water and energy coupling during 1979–2020.

Further analysis shows that the decadal variation in EF can be used to qualitatively indicate the decadal variation in ET, which is of general interest. Conventional methods for estimating the ET variation require a large number of inputs, some of which in fact lack reliable data. Differences in the input data sources used for ET products can lead to significant differences in decadal variations between different ET products. We find a high positive correlation between EF and ET on the decadal scale, which suggests that changes in ET can be characterized by changes in EF. Since the EF calculation based on SFE theory only requires inputs of near-surface air temperature and specific humidity, this relationship substantially reduces the difficulty of estimating long-term changes in ET and provides a new idea for water cycle studies.

In summary, the approach of calculating EF based on local SFE theory is simple and its input data (near-surface air temperature and specific humidity) are easily accessible. Since this approach can be used to calculate the decadal variability of EF and ET even on the Tibetan Plateau where convective activities are vigorous, it is expected that this approach can be extended to other regions of the globe to study the decadal and longer-term variability of water and energy coupling.

## Declaration of competing interest

The authors declare that they have no conflicts of interest in this work.
